# Left Ventricular Mass Index in End-Stage Renal Disease Patients during Hemodialysis and Continuous Ambulatory Peritoneal Dialysis

**DOI:** 10.1155/2023/8816478

**Published:** 2023-12-11

**Authors:** Nghia Nhu Nguyen, Phieu Van Duong, Tan Huynh Ngoc Mai, Nghia Hoang Vo, Dinh Kim Luong, Toan Hoang Ngo

**Affiliations:** ^1^Department of Internal Medicine, Faculty of Medicine, Can Tho University of Medicine and Pharmacy, Can Tho 900000, Vietnam; ^2^Department of Internal Medicine, Faculty of Medicine, Nam Can Tho University, Can Tho 900000, Vietnam

## Abstract

**Background:**

One of the primary reasons for high mortality in end-stage renal disease (ESRD) is cardiovascular disease in patients with renal replacement therapy (RRT). Left ventricular hypertrophy (LVH) significantly predicts mortality and cardiovascular events.

**Objectives:**

We assess the left ventricular mass index change in two dialysis methods: hemodialysis (HD) and continuous ambulatory peritoneal dialysis (CAPD). The factors associated with increased left ventricular mass index (LVMI).

**Materials and Methods:**

We recruit more than 50 HD patients and 45 CAPD patients with LVH of similar age, gender, dialysis duration, and LVMI for one-year follow-up.

**Results:**

The LVMI in the group of HD patients after one year increased from 180.28 ± 45.32 g/m^2^ to 212.58 ± 66.22 g/m^2^ (*p* = 0.001), while the LVMI in the group of patients with CAPD increased from 190.16 ± 66.01 g/m^2^ to 197.42 ± 78 g/m^2^ (*p* = 0.32). Multivariable logistic regression analysis, we demonstrated that dialysis by HD (*β* = −1,167, 95% CI: 0.104–0.938, *p* = 0.036) and anemia treatment lower the goals (*β* = 1.9566, 95% CI: 1.466–34.094, *p* = 0.015) were two factors associated with the progression of the LVMI.

**Conclusion:**

The LVH of end-stage renal disease patients with HD treatment is worse than CAPD treatment after a follow-up in one year. Dialysis by periodic hemodialysis and anemia treatment that fails to achieve the goal are risk factors associated with increased progression of LVMI in patients with ESRD.

## 1. Introduction

Chronic kidney disease (CKD) has been recognized as a leading public health problem worldwide. The global estimated prevalence of CKD is 13.4% (5–15%), and patients with end-stage kidney disease (ESKD) who require renal replacement therapy (RRT) are estimated between 5 and 7 million [1]. Left ventricular hypertrophy (LVH) is present in 15–21% of the general population but affects 50–70% of patients with CKD and as many as 80% of patients with CKD on dialysis [2]. LVH also has been associated with the ambulatory arterial stiffness index (AASI) and left ventricular mess index (LVMI) [3]. In some studies, authors reveal that the progression of LVH in patients with ESRD is associated with mortality and incident cardiovascular events [4].

The analysis of previous research has demonstrated that cyclic continuous ambulatory peritoneal dialysis is superior to periodic hemodialysis for maintaining cardiovascular function. However, how these procedures affect the left ventricular mass index (LVMI) is unclear. We conducted this study to assess changes in the left ventricular muscle mass index of two methods: hemodialysis (HD) and continuous ambulatory peritoneal dialysis (CAPD), and find some factors related to the change in LVMI.

## 2. Materials and Methods

### 2.1. Study Population

#### 2.1.1. Materials

157 patients with end-stage chronic kidney disease with indications for renal replacement therapy at Can Tho City General Hospital from February 2021 to May 2022 were included in the study.

#### 2.1.2. Inclusion Criteria

We performed an echocardiographic examination on 157 patients with ESKD who require dialysis (eGFR <15 ml/minutes/1.73 m^2^), then excluded patients without LVH according to Framingham criteria [5] and randomized them into two groups: Hemodialysis and continuous ambulatory peritoneal dialysis. Participants were similar in age, sex, dialysis duration, and baseline LVMI. These patients aged 18 years and older were treated with periodic hemodialysis three times/week (9–12 hours/week) with localization vascular access of arteriovenous fistula is brachial artery or peritoneal cycle dialysis four times/day (2 liters per day) with a dialysis duration of at least three months.

#### 2.1.3. Exclusion Criteria

Patients with the following exclusion criteria: patients with acute diseases such as severe infections, acute coronary syndromes, acute heart failure, cerebral infarction, or cerebral hemorrhage; patients receiving treatment who pass away or are transferred to another medical facility; patients undergoing renal transplantation or continuous ambulatory peritoneal dialysis are converted to hemodialysis; patients who declined to participate in the study.

### 2.2. Methods

#### 2.2.1. Study Design

A cross-sectional descriptive study with a longitudinal follow-up was conducted.

#### 2.2.2. Sample Size

**Figure 1:**
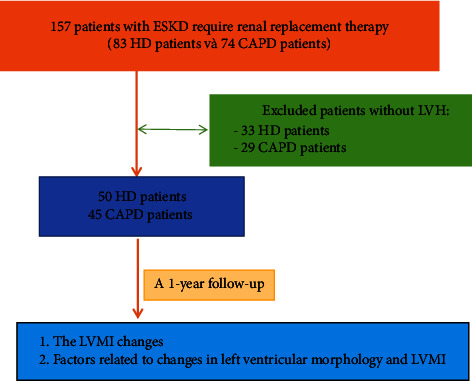
Participant flow diagram.

All patients who met the sampling criteria had a clinical examination, laboratory examination, and first echocardiography to evaluate left ventricular morphological indices and left ventricular muscle mass index (LVMI); in HD patients, the echocardiograms were performed after the dialysis session on the day between 2 weekly dialyzes, and for CAPD patients, after the first fluid change of the day when the patient visited the CAPD management clinic. Then, we selected from two groups of HD and CAPD patients with left ventricular hypertrophy similar regarding age, sex, dialysis duration, and LVMI. Patients in both groups continued to receive renal replacement therapy with the above two methods; patients continued to obtain background medical care, treated with RRT, controlled for blood pressure (BP), erythropoietin for anemia, and statins for dyslipidemia. All patients were asked for medical history, history, physical examination, height, and weight measurements (before dialysis for patients with HD and at monthly follow-up for patients with CAPD). The dialysis indices represent the mean values averaged across the various dialysis sessions; hematological and biochemical blood tests are performed every three months. After 12 months, we assessed the left ventricular morphology and LVMI for the second time in these patients' clinical, subclinical, and echocardiographic records (Figure 1).

### 2.3. Data Collection

#### 2.3.1. Data Analysis

Left ventricular hypertrophy is defined as LVMI >131 g/m^2^ in male patients and LVMI >100 g/m^2^ in female patients according to Framingham criteria, concentric left ventricular hypertrophy when there is left ventricular hypertrophy and RWT ≥0.42, and eccentric left ventricular hypertrophy when there is left ventricular hypertrophy and RWT <0.42, and RWT is the calculated relative wall thickness according to the formula RWT = 2xLVPWd/LVIDd [5]. In liters (L), the daily urine volume measures the urine produced in 24 hours. The goal of hemoglobin for CKD patients treated with erythropoietin is 10–11.5 g/dL [6], and the target is not met when hemoglobin is less than 10 g/dL, according to the KDIGO Guidelines (2012). A smoker is an adult who has smoked 100 cigarettes in his or her lifetime and currently smokes cigarettes, according to the COMMIT criteria (Community Intervention Trial). Never smoker: An adult who has never smoked or has not smoked in the past five years [7]. The progression of left ventricular mass index was defined as higher than the mean of ∆LVMI (∆LVMI = LVMI at one year-baselines LVMI).

#### 2.3.2. Measurements

The left ventricle (LV) structure was assessed by Philips HD11 echocardiogram with a Phased Array transducer orientation and transducer position for the parasternal long axis and the apical four-chamber view its modifications. We used the M-mode echocardiography for the parasternal long axis to measure: IVSs, IVSd, LVIDd, LVIDs, LVPWd, and LVPWs. Our LVMI calculator uses the following equation LVM (g) = 0, 8x (1, 04x(LVIDd + IVSd + LVPWd)³ − LVIDd³)) + 0, 6 [8]. LVDd is the left ventricular end diastolic diameter, IVSd is the inter ventricular septal thickness end diastolic, and LVPWd is the left ventricular end diastolic post wall. LVMI is the short term for the LV mass indexed to body surface area (LVMI = LVM/BSA) [8].

### 2.4. Statistical Analysis

The data processing method using SPSS 20.0 software presents qualitative variables by frequency and percentage. Continuous quantitative variables are presented as the mean ± standard deviation, minimum value, and maximum value. The chi-square test is applied to investigate the relationship between ratios (Extract's Fisher correction in the case of a 2 × 2 table with at least one expected value <5). *T*-test was used to investigate the difference in mean between the two groups. Paired *T*-test is applied to investigate whether the difference in mean values before and after treatment intervention of the two groups is significant. Multivariable logistic regression evaluates the relationship between the dependent variable (a qualitative variable) and the independent variable (a qualitative or quantitative variable).

## 3. Results

### 3.1. Baseline Subject Characteristics

There was no difference in age, gender, dialysis duration, and background medical history between hemodialysis and continuous ambulatory peritoneal dialysis patients. Regarding subclinical, the mean albumin levels of the CAPD group were lower than that of the HD group (*p*  <  0.05) and all patients undergoing hemodialysis have an arteriovenous fistula (AVF) site in the brachial artery (Table 1). Initially, there was a nonsignificant difference in the echocardiogram of both groups (Table 2).

### 3.2. Factors Related to Changes in Left Ventricular Morphology and LVMI on Echocardiography in 2 Groups HD and CAPD after One Year of Follow-Up

**Figure 2:**
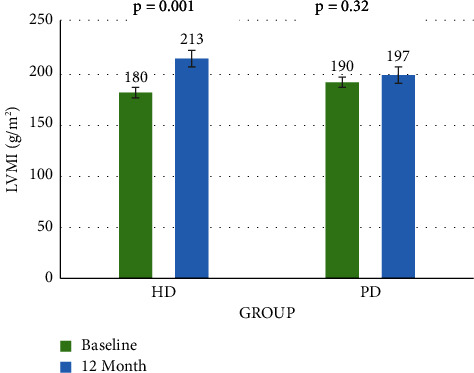
Mean left ventricular mass index (LVMI) from baseline to 12 months.

After 12 months, at the second echocardiogram, there was no dramatic change in the morphological index of the CAPD group. In contrast, in the HD group, the IVSs, LVPWd, and LVPWs increased significantly compared to the first echocardiogram (Table 3). LVMI substantially increased in the group receiving periodic hemodialysis (*p* = 0.001). The LVMI remained unchanged in the continuous ambulatory peritoneal dialysis group at 12 months (*p* = 0.32). The left ventricular mass index of the CAPD group did not change much from 190.16 ± 66.01 g/m^2^ at the baseline to 197.42 ± 78 g/m^2^ after one year (*p*  >  0.05), an average increase of 7.25 g/m^2^. In contrast, the LVMI in the HD group increased significantly from 180.28 ± 45.32 g/m^2^ to 212.58 ± 66.22 g/m^2^(*p*  <  0.05), an average increase of 32.29 g/m^2^ after one year (Figure 2). Multivariate logistic regression analysis to assess factors related to the progression of LVMI in ESRD patients on dialysis: Under-targeted anemia treatment and hemodialysis treatment were predictors of left ventricular mass index (LVMI) progression in ESRD patients on dialysis (Table 4), (Table 5).

## 4. Discussion

### 4.1. Baseline Subject Characteristics

Regarding the age of the study subjects, when compared with other studies, we found that the ESRD patients in Vietnam were younger than the ESRD patients in the world. Tian et al. (China) observed that the middle-age average is 60.2 ± 11.0 (CAPD) and 58.8 ± 13.6 (HD) [9]. Similarly, in Korea, Jung et al. also found the mean age of HD and CAPD patients as 56.6 ± 13.5 and 51.6 ± 12.8, respectively [10].

### 4.2. Changes of Left Ventricular Morphological Indexes and LVMI, and Factors Related to Changes in Left Ventricular Morphology and Left Ventricular Mass Index on Echocardiography in 2 Groups: HD and CAPD, after One Year of Follow-Up

When analyzing the multivariable logistic regression, the results indicated that factors such as hemodialysis treatment and anemia treatment were not associated with the progression of left ventricular mass index in patients with end-stage renal disease undergoing dialysis. The continuous ambulatory peritoneal dialysis method holds advantages over HD in terms of cardiovascular function. Hemodialysis patients who require arteriovenous fistula placement often experience volume overload due to increased venous return to the heart, leading to heightened cardiac load and severe left ventricular hypertrophy. Hemodialysis requires three sessions per week, with a total dialysis duration of 9–12 hours, while patients in the CAPD group undergo continuous peritoneal dialysis, leading to frequent fluid and substance exchanges. This contributes to a higher rate of ultrafiltration in the CAPD group compared to the HD group (0.89 ± 0.42 vs. 0.37 ± 0.04, *p*  <  0.001). When comparing our results with various longitudinal studies examining left ventricular hypertrophy and muscle mass in HD patients, our findings align with the observations of Zoccali et al. [4] and Moon et al. [11]. Our study's insignificant change in left ventricular mass over the one-year follow-up period resonates with findings reported in other studies [12, 13].

Anemia stands as an independent risk factor for left ventricular hypertrophy, heightened hospitalization rates, and mortality among dialysis patients. The increase in mortality is particularly prominent when hemoglobin (Hb) levels fall below or equal to 8 g/dL [14]. Several investigations have demonstrated a correlation between low hemoglobin levels, specifically Hb levels below 7.7 g/dL, and an augmentation in left ventricular muscle mass [14]. Silberberg et al. observed that with each 1 g/dL reduction in Hb, the pace of pulmonary parenchymal transit (PPT) escalated by 6% [15]. In cases of chronic anemia, the cardiovascular system adapts to counter the diminished capacity to supply oxygen to the body. This adaptation leads to heightened cardiac output and peripheral vasodilation in response to hypoxia. The resultant vasodilation, in conjunction with reduced blood viscosity, contributes to decreased peripheral resistance. The sustained elevation in cardiac output induces a compensatory augmentation in left ventricular mass. Several global studies also highlight the significant impact of anemia treatment on the left ventricular mass index. Levin et al. examined 226 chronic renal failure patients and found that a decrease of 0.5 g/dL in Hb led to a 1.32-fold increase in the left ventricular mass index during the second echocardiogram conducted one year after baseline (*p* = 0.004) [16]. Drawing from the study's outcomes, we propose that dialysis patients should receive aggressive anemia treatment to achieve the targeted Hb levels.

### 4.3. Limitations

Indeed, our study has certain limitations that warrant acknowledgment. Primarily, the follow-up duration was relatively brief, encompassing just one year. Additionally, the evaluation of factors associated with the progression of the left ventricular mass index, including factors like fluid overload, was not executed in a comprehensive manner. Moreover, the absence of longitudinal follow-up spanning 45–47 months at the culmination of the dialysis cycle has left certain aspects unexplored. Furthermore, it is important to note that all participants in our study were subjected to a single access method, either fistula or peritoneal dialysis, within a singular cohort. This design parameter inevitably restricts the generalizability of our findings to this specific context. Considering these limitations, we recommend that future research endeavors consider a more expansive sample size, the incorporation of a randomized and double-blind study design, an extended follow-up period, and a more comprehensive evaluation of factors contributing to the progression of left ventricular hypertrophy.

## 5. Conclusion

After one year, the left ventricular mass index significantly increased in hemodialysis (HD) patients with end-stage chronic renal disease, but not in continuous ambulatory peritoneal dialysis (CAPD) patients. In comparison to the CAPD patient group, left ventricular hypertrophy in HD patients worsened over time. Multivariate logistic regression analysis indicated that under-targeted anemia treatment and hemodialysis treatment are predictors of LVMI progression in ESRD patients undergoing dialysis.

## Figures and Tables

**Table 1:** Baseline characteristics of the study population.

Parameter	HD (*n* = 50)	CAPD (*n* = 45)	*p*
Age (years)	51.3 ± 10.33	47.24 ± 12.17	*p* = 0.055
Male (*n*, %)	22 (52.4%)	20 (47.6%)	*p* = 0.965
Duration dialysis (months)	47.26 ± 28.69	44.56 ± 29.69	*p* = 0.653
Hypertension (*n*, %)	50 (100%)	45 (100%)	*p* > 0.05
Diabetes mellitus (*n*, %)	10 (20%)	7 (15.6%)	*p* = 0.573
ACD (*n*, %)	40 (80%)	28 (62.2%)	*p* = 0.055
Smoking (*n*, %)	18 (36%)	14 (31.1%)	*p* = 0.615
BMI (kg/m^2^)	21.53 ± 2.57	21.92 ± 4.05	*p* = 0.583
SBP (mmHg)	140.50 ± 12.62	143.78 ± 17.09	*p* = 0.295
DBP (mmHg)	84 ± 8.08	84 ± 8.63	*p* > 0.05
Antihypertensive drugs			
ACEI/ARB (*n*, %)	48 (96%)	37 (82.2%)	*p* = 0.043
CCB (*n*, %)	47 (94%)	42 (93.3%)	*p* = 0.61
ΒB (*n*, %)	43 (86%)	41 (91.1%)	*p* = 0.437
Diuretics (*n*, %)	28 (56%)	26 (57.8%)	*p* = 0.861
Methyldopa (*n*, %)	32 (64%)	22 (48.9%)	*p* = 0.138
24 h urine volume (ml)	181 ± 187	331 ± 344	*p* = 0.025
UV (L)	0.37 ± 0.04	0.89 ± 0.42	*p* < 0.001
eGFR	4.54 ± 1.32	5.52 ± 1.64	*p* < 0.05
Dyslipidemia (*n*, %)	33 (66%)	29 (64.4%)	*p* = 0.874
Statin (*n*, %)	43 (86%)	34 (75.6%)	*p* = 0.195
Hb (g/dL)	9.16 ± 1.55	9.74 ± 1.32	*p* = 0.055
Erythropoietin (*n*, %)	47 (94%)	44 (97.8%)	*p* = 0.619
Albumin (g/L)	38.3 ± 3.58	30.29 ± 4.29	*p* < 0.001
Calcium (mmol/L)	1.86 ± 0.31	1.99 ± 0.22	*p* < 0.05
Phosphate (mmol/L)	1.84 ± 0.58	1.78 ± 0.45	*p* = 0.568
Cholesterol (mmol/L)	4.13 ± 0.97	4.55 ± 1.16	*p* = 0.061
LDL-c (mmol/L)	3.03 ± 1.04	3 ± 1.18	*p* = 0.089
HDL-c (mmol/L)	1.06 ± 0.24	1.17 ± 0.21	*p* < 0.05
TG (mmol/L)	1.95 ± 1.17	1.85 ± 1.14	*p* = 0.681

ACD, atherosclerotic cardiovascular disease, ACEi, angiotensin-converting enzyme inhibitors; ARB, angiotensin receptor blockers; BMI, body mass index; CCB, calcium channel blockers; DBP, diastolic blood pressure; SBP, systolic blood pressure; BB, beta blockers; Hb, hemoglobin; LDL-c, low density lipoprotein cholesterol; HDL-c, high density lipoprotein cholesterol; TG, triglyceride; UV, ultrafiltration volume.

**Table 2:** Reference values for echocardiographic measurements in HD patients and CAPD patients.

Parameter	HD (*n* = 50)	CAPD (*n* = 45)	*p*
IVSd (mm)	13.76 ± 2.52	13.95 ± 2.59	*p* = 0.71
IVSs (mm)	16.16 ± 2.4	16.77 ± 3.01	*p* = 0.27
LVIDd (mm)	46.69 ± 5.47	50.31 ± 6.78	*p* = 0.62
LVIDs (mm)	32.87 ± 4.52	33.25 ± 7.15	*p* = 0.75
LVPWd (mm)	13.17 ± 2.03	13.51 ± 2.99	*p* = 0.51
LVPWs (mm)	16.64 ± 2.53	17.76 ± 3.21	*p* = 0.06
RWT (cm)	0.53 ± 0.11	0.54 ± 0.13	*p* = 0.8
LVM (g)	275.92 ± 68.93	295.69 ± 111.49	*p* = 0.3
LVMI (g/m^2^)	180.28 ± 45.32	190.16 ± 66.01	*p* = 0.4
EF (%)	62.13 ± 7.92	62.28 ± 10.61	*p* = 0.93

IDSs, interventricular septal systolic; IDSd, interventricular septal diastolic; LVIDd, left ventricular internal diameter diastolic; LVIDs, left ventricular internal diameter systolic; LVPWd, left ventricular posterior wall diastolic; LVPWs, left ventricular posterior wall systolic; LVMI, left ventricular mass index; LVM, left ventricular mass; RWT, relative wall thickness; EF, ejection fraction.

**Table 3:** Changes of left ventricular morphological indexes and LVMI after one year in two groups of HD and CAPD patients.

Parameter	Follow up time	HD	CAPD
IVSd	Baseline	13.76 ± 2.52	13.95 ± 2.59
	After 12 months	14.59 ± 3.42	13.45 ± 2.92

IVSs	Baseline	16.16 ± 2.4	16.77 ± 3.01
	After 12 months	18.36 ± 3.16^*∗*^	17.35 ± 3.43

LVIDd	Baseline	46.69 ± 5.47	50.31 ± 6.78
	After 12 months	50.64 ± 7.62	50.87 ± 8.08

LVIDs	Baseline	32.87 ± 4.52	33.25 ± 7.15
	After 12 months	32.58 ± 6.25	33.74 ± 8.06

LVPWd	Baseline	13.17 ± 2.03	13.51 ± 2.99
	After 12 months	15.19 ± 2.23^*∗*^	14.29 ± 3.7

LVPWs	Baseline	16.64 ± 2.53	17.76 ± 3.21
	After 12 months	18.84 ± 2.71^*∗*^	17.76 ± 3.01

LVM	Baseline	275.92 ± 68.93	295.69 ± 111.49
	After 12 months	332.85 ± 107.44^*∗*^	307.49 ± 132.1

LVMI	Baseline	180.28 ± 45.32	190.16 ± 66.01
	After 12 months	212.58 ± 66.22^*∗*^	197.42 ± 78

EF	Baseline	63.82 ± 8.41	61.77 ± 12.24
	After 12 months	62.13 ± 7.92	62.28 ± 10.61

Paired *T*-test. IDSs, interventricular septal systolic; IDSd, interventricular septal diastolic; LVIDd, left ventricular internal diameter diastolic; LVIDs, left ventricular internal diameter systolic; LVPWd, left ventricular posterior wall diastolic; LVPWs, left ventricular posterior wall systolic; LVMI, left ventricular mass index; LVM, left ventricular mass; EF, ejection fraction; ^*∗*^*p*  <  0.05.

**Table 4:** Comparison of the mean values of certain parameters during a one-year follow-up between the group with progressing LVMI and the group with nonprogressing LVMI.

Parameter	Progression LVMI	No progression LVMI	*p*
ACEI/ARB therapy (%)	29.4%	70.4%	*p* < 0.05
Statin therapy (%)	26%	74%	*p* < 0.05
UV (L)	0.54 ± 0.29	0.68 ± 0.35	*p* = 0.06
SDP (mmHg)	143.92 ± 12.27	138.05 ± 15.92	*p* = 0.1
DDP (mmHg)	86.07 ± 12.27	82.76 ± 12.1	*p* = 0.23
Hb (g/dL)	8.50 ± 1.35	9.45 ± 1.61	*p* < 0.05
Cholesterol (mmol/L)	4.35 ± 1.33	4.51 ± 1.27	*p* = 0.57
LDLc (mmol/L)	2.88 ± 1.12	3.03 ± 1.11	*p* = 0.56
Albumin (g/dL)	32.71 ± 2.73	32.58 ± 4.38	*p* = 0.88

ACEi, angiotensin-converting enzyme inhibitors; ARB, angiotensin receptor blockers; UV, ultrafiltration volume; DBP, diastolic blood pressure; SBP, systolic blood pressure; Hb, hemoglobin; LDL-c, low density lipoprotein cholesterol.

**Table 5:** Multivariable logistic regression analysis of factors that associated with the progression of the left ventricular mass index.

Parameter	Multivariable analysis		
	*β*	95% CI	*p*
Hemodialysis	−1.167	0.104–0.938	*p* = 0.036
Hb treatment not achieving targe	1.956	1.466–34.094	*p* = 0.015
ACEI/ARB therapy	0.486	0.317–8.339	*p* = 0.56
Statin therapy	1.183	0.939–11.342	*p* = 0.063

## Data Availability

The datasets generated and/or analyzed during the current study are available from the corresponding author on reasonable request.
